# Integration of Sex and Gender into Health Professions Education

**DOI:** 10.1089/jwh.2019.8117

**Published:** 2019-12-10

**Authors:** Alyson J. McGregor, Marjorie Jenkins

**Affiliations:** ^1^ Division of Sex and Gender in Emergency Medicine, Warren Alpert Medical School of Brown University, Providence, Rhode Island.; ^2^ Texas Tech University Health Sciences School of Medicine, Laura W. Bush Institute for Women's Health, Lubbock, Texas.

Science demonstrates the influence that sex as a biological variable and gender as a sociocultural variable have in prevention, screening, diagnosis, treatment, and outcomes of health and disease in women and men. Results from a national medical student survey showed unequivocally that medical students wanted this knowledge integrated into their curricula.^1^ We must act to improve the education of future health professionals by imbuing them with the necessary knowledge and skills to deliver sex and gender-specific clinical care.

As scientific discovery continues to elucidate the many ways sex and gender influence patient care and outcomes coupled with the desire health care learners have to know more, curricular transformation is no longer a choice but a requirement. The push for integrating sex and gender into health professions curricula began in 2012 at a workshop held at the Mayo Clinic. Representatives from medical schools across the United States gathered to discuss strategies to achieve integration. After this meeting, the first Sex and Gender Medical Education (SGME) Summit was held in 2015 at the Mayo Clinic. Stakeholders included representatives from >150 U.S. and international medical schools as well as representatives from federal agencies and nonprofit organizations.

The next step was for interprofessional teams to work together to progress the integration of sex and gender content into all health professions curricula. The Sex and Gender Health Education (SGHE) Summit became that historic event and the first time that the five major U.S. health professions—medicine, nursing, dentistry, pharmacy, and allied health—convened at the University of Utah for the purpose of progressing SGHE on a national scale with >250 health care professionals from 162 institutions, health care centers, organizations, and agencies in attendance.

The goals for the SGHE Summit were to create a network of health professionals and health educators that can support and progress the curricular integration of scientific evidence surrounding the impact of sex and gender on health. Basic tenets of sex and gender health were presented and explored within the framework of the different health professions. Three central questions form the main themes for the SGHE Summit:

- What sex and gender content need to be incorporated into curricula to prepare our students for the future?
- What is the best way to do this?
- What can you do now to accelerate transformation?

Resources provided at the summit were designed to review current health professions' accreditation standards in sex and gender, facilitate curricular transformation through strategies that assess home institutions state of readiness for change, create an action plan that fits each organization and health profession as well as utilize existing tools and resources. Faculty development and a team-based approach is key. Attendees were provided with ready-made peer-reviewed sex and gender educational resources provided by the Laura W. Bush Institute.

Importantly, the information shared was delivered with a solid foundation of educational theory, accreditation requirements, and interprofessional models. The engagement, conversations, and feedback at the SGHE Summit have been incredible. An example of a collaborative effort to create a true interprofessional community where we learn from and support each other.

As guest editors we are pleased to showcase the SGHE Summit proceedings and workshop outcomes in this special issue of the *Journal of Women's Health* on “Integration of Sex and Gender into Health Professions Education.” We are also thrilled to provide a platform to include additional articles on various approaches of sex and gender knowledge dissemination. This collection provides strategies for incorporating sex and gender-based health content into existing curricula to fill knowledge gaps and achieve sustainable change.
